# The relationship between memory complaints and age in normal aging

**DOI:** 10.1590/S1980-57642009DN30200005

**Published:** 2009

**Authors:** Thaís Bento Lima-Silva, Mônica Sanches Yassuda

**Affiliations:** 1 Undergraduate student in Gerontology at EACH-USP, São Paulo, SP, Brazil.; 2 PhD, Assistant professor of Gerontology at EACH-USP, São Paulo, SP, Brazil.

**Keywords:** memory complaints, memory, age, elderly, cognition

## Abstract

Normal aging can be characterized by a gradual decline in some cognitive functions, such as memory. Memory complaints are common among older adults, and may indicate depression, anxiety, or cognitive decline.

**Objectives:**

To investigate the association between memory complaints and age in cognitively unimpaired older adults, and the relationship between memory complaints and memory performance.

**Methods:**

Cognitive screening tests as well as memory complaint questionnaires validated for the Brazilian population were used: the Mini-Mental State Examination (MMSE), Geriatric Depression Scale (GDS), Memory Complaint Questionnaire (MAC-Q), Memory test of 18 pictures, Forward and Backward Digit Span (WAIS-III). Fifty seven regular members of the SESC social club participated (50 women), having a mean age of 71.4 years, and 4 to 8 years of education - 34 from 4 to 7 years and 23 with 8 years of education.

**Results:**

Results revealed no significant association between cognitive complaints and age or cognitive performance. Older participants in this sample did not show worse performance or a higher level of complaints. There was no significant association between age and GDS scores.

**Conclusions:**

The studied sample constitutes a particular group of older adults whose participation in activities may be protecting them from cognitive decline, thus highlighting the impact of lifestyle on cognitive performance during the aging process.

## Abstract (pt)

O envelhecimento normal caracteriza-se por um declínio gradual em algumas funções cognitivas, como a memória. As queixas de memória são comuns entre os idosos, podendo sinalizar depressão, ansiedade, ou declínio cognitivo.

**Objetivos:**

Verificar a relação entre a queixa de memória e a idade no envelhecimento normal e a relação da queixa com o desempenho cognitivo.

**Métodos:**

Foram aplicados testes de rastreio cognitivo e escalas de queixas validados para a população idosa brasileira: Mini-Exame do Estado Mental (MEEM), Escala de Depressão Geriátrica (GDS), Questionário de Queixas de Memória (MAC-Q), Escala de freqüência de esquecimentos, Teste de memorização de 18 figuras, Dígitos ordem direta e inversa (WAIS-III). Participaram 57 freqüentadores do SESC (50 mulheres), com idade média de 71,4 anos, e escolaridade entre 4 e 8 anos, 34 com Ensino Fundamental incompleto (entre 4 e 7 anos), e 23 com Ensino Fundamental completo.

**Resultados:**

Não houve associação significativa entre queixa de memória, idade e desempenho cognitivo. Os participantes mais velhos não apresentaram pior desempenho ou maior número de queixas de memória. Não houve associação entre idade e GDS.

**Conclusões:**

A amostra estudada pode constituir um grupo particular de idosos, entre os quais as atividades desenvolvidas poderiam atuar como fator de proteção da cognição, destacando a possiblidade do estilo de vida influenciar o desempenho cognitivo durante o processo de envelhecimento.

Memory complaints are known to be part of the meta-memory construct.^1^ This construct refers to knowledge and beliefs held about memory. It encompasses the knowledge we have on the mnemonic demands imposed by different tasks and situations, as well as the knowledge about the strategies which may be employed to enhance memory efficiency.^2^

Complaints and concerns regarding memory are present in the population at large but tend to become more prevalent with increasing age.^3^ Many studies have investigated the significance of memory complaints in older adults and principally, have sought to identify the existence of an association between subjective perception of memory loss and objective impairment verified by objective testing.^4,5^ The association between objective memory (performance on memory tasks) and subjective memory (complaints, perceptions, affects, beliefs about memory) has been the subject of study for at least 30 years. Moreover, the extent to which memory complaints are predictive of age-associated cognitive decline and dementia onset has been investigated in the clinical context.

A broad range of studies on the association between subjective and objective memory are available in the international literature. These findings, however, remain controversial. The majority of cross-sectional studies have found no strong association between memory complaints and performance.^6^ Nevertheless, several cross-sectional studies showing a positive association between complaints and cognitive decline are worthy of note.

Jonker and colleagues^7^ conducted a study involving 2367 patients without dementia or depressive symptoms and found a strong association between memory complaints and poor performance on the animal category verbal fluency test, even after adjusting for age, gender and verbal intelligence.

A significant correlation between memory complaints and the presence of depressive symptoms has also been reported in cross sectional studies – a relationship which appears to be stronger than the association between complaints and cognitive performance.^4,8-10^ Studies such as that the one conducted by Hanninen and colleagues,^11^ have reported that individuals with cognitive complaints have a greater propensity to present somatic complaints, as well as a greater feeling of incompetence and incapacity than non-complainers. These findings reveal that complaints are linked to other variables. For example, Van Oijen and colleagues^12^ showed that the association between memory complaints and risk of developing Alzheimer’s disease varied according to educational strata, there being a stronger link among more highly educated individuals. Thus, memory complaints among highly schooled individuals could represent a significant indicator of dementia onset.

Longitudinal studies frequently suggest that complaints are predictive of cognitive decline. As reported by Schofield and colleagues,^13^ complaints may be more informative if reported by individuals who do not present the profile of a complainer. These authors studied patients who had previously denied having a problem and who subsequently reported complaints. After a one year period, 26% of initial non-complainers had gone on to report memory complaints. These recent complainers had a five-fold higher risk of developing mild cognitive impairment than those who remained with no complaints.

Dik and colleagues^14^ conducted a six-year follow-up study in a cohort of patients, and the presence of complaints was associated with cognitive decline in two out of the four cognitive tests used. Patients with complaints performed worse than non-complainers on both the letter substitution test and the immediate recall test of the Rey Auditory Verbal Learning Test (RAVLT). However, performance on the delayed recall of the RAVLT and on the Mini-Mental State Exam (MMSE) was similar for both complainers and non-complainers.

The sample followed by Palmer and colleagues^15^ included a proportion of individuals with cognitive decline but without dementia, characterized by scores on the MMSE greater than or equal to 20. These authors found a positive correlation between complaints and progression to dementia after three and a half years. It should be emphasized that their statistical analysis was not adjusted to compensate for possible differences in dementia prediction due to cognitive status at study baseline.

In Brazil, the study by Mattos and colleagues^16^ compared the use of a self-report structured questionnaire versus direct questioning about memory problems in a sample of healthy community-dwelling elderly persons (63 women) without risk factors for cognitive deficits. Participants were asked about subjective memory complaints (SMC), submitted to the Memory Complaints Questionnaire (MAC-Q) and the Rey Auditory Verbal Learning Test (RAVLT). It was noted that although the presence of complaints was correlated with higher scores on the MAC-Q, a significant percentage of the sample obtained low scores on this instrument despite having presented with complaints. Moreover, some individuals without memory complaints scored highly on the MAC-Q. Performance on the RAVLT was significantly worse in the group reporting complaints during direct questioning (p<0.05), but not in the group with high scores on the MAC-Q. Therefore, direct questioning about complaints may be clinically more valuable than a self-report questionnaire, at least among community-dwelling elderly without risk factors for cognitive deficit or depression.

Almeida^17^ detected memory complaints in 7% of patients in a mental health outpatient unit (31 out of 220 elders). A study by Minett and colleagues^6^ investigated the significance of memory complaints in a Brazilian geriatric clinic and their predictive value for detecting dementia. The percentage of older adults with memory complaints was 21%. The authors stressed that this result was chiefly due to the methodology employed, in which elders who deemed that their memory problems interfered with daily tasks were considered complainers. Significant differences between complainers and non-complainers were found only for the verbal fluency test and the Geriatric Depression Scale (GDS). According to Minett and colleagues,^6^ the frequency of memory complaints found among older adults can vary substantially depending on the study sample profile, being higher in community-based studies which typically include a higher proportion of older elderly than studies involving volunteers.

Amid this context of controversial findings, the aim of the present study was to investigate the relationship between the age and the presence of memory complaints. It is known that some cognitive functions become significantly compromised with aging. Therefore, we tested the hypotheses that older elderly present more frequent memory complaints, and that a greater number complaints are associated to worst cognitive performance on objective memory tests.

## Methods

A total of 67 elderly individuals were recruited, aged fifty six years and older, given that memory complaints tend to emerge more frequently after fifty. Participants had between 4 to 8 years of schooling and were active members of SESC. The range of schooling years was restricted in order to reduce any effect of age on complaints and cognitive results. Participants were divided into two age groups, Group 1 aged between 56 and 74 years, and Group 2 aged between 75 and 92 years.

All subjects underwent an initial assessment composed of a socio-demographic questionnaire, and screening tests to detect possible presence of depression and/or dementia. Elderly who presented signs of the pathologies outlined above were not interviewed according to the exclusion criteria of the study, which were being younger than 55 years of age, presenting signs of depression or cognitive decline, and presenting auditory or visual decline that prevented conducting the interview.

The recruited participants all signed a free and informed consent term. Participants were subsequently submitted to a pre-assessment using the Geriatric Depression Scale. A cut-off point of 5, initially proposed by Almeida and Almeida,^18^ was adopted in the present study, whereby older adults scoring 6 or more on the GDS were not included in the study.

Dementia screening entailed application of the Mini-Mental State Exam proposed by Brucki and colleagues,^19^ where reported medians were adopted as the cut-off points, namely, a score of 25 for older adults with up to 4 years of schooling, and 26.5 for older adults with 5 to 8 years of schooling.

The Memory Complaints Questionnaire – MAC-Q^16,20^ was used to assess memory complaints. The MAC comprises six questions assessing memory complaints in five everyday situations, for example, recalling telephone numbers or codes which are used daily or weekly, recall of items from a shopping list, and one question addressing global mnemonic performance. Subjects were asked to compare current mnemonic performance with that at 18 years of age. Responses ranged from “much worse now” to “much better now”, with five different possible answers. Scores greater than or equal to 25 points were considered indicative of subjective memory impairment,^16,21^ with a maximum score of 30 points.

An adapted version of the scale by McNair and Kahn^23^ of frequency of forgetfulness was also used. Based on this scale, participants indicated how often they forgot passwords, peoples’ names, among other commonly forgotten items, selecting Never (0), Sometimes (1), Frequently (2), or Always (3). Scores range from 0 to 45 points on this scale, with higher scores denoting greater frequency of memory complaints or forgotten items. To date, no studies have established a cut-off point, for frequency of forgotten items, which would be indicative of clinically significant memory deficits.

The scale proposed by Carvalho,^24^ containing 18 common black and white pictures categorized into three different semantic groups, was used to assess episodic memory. Participants were given one minute to examine the pictures. Subsequently, participants carried out a five-minute distractor task in the form of the Forward and Backward Digit Span test from the WAIS-III battery.^25^ This requires the interviewee to repeat several sequences of digits in forward and backward direction. The total score is the sum of the correctly repeated sequences of 16 and 14 points, respectively. Upon task completion, the interviewee returned to the picture task and recalled the name of as many pictures studied as possible.

### Statistical analysis

Frequency tables of categorical variables (gender, schooling, personal income and current occupation) were built to provide a profile of the sample. Descriptive statistics (mean, standard deviation, minimum and maximum values) of the continuous variables were calculated, including scores on the cognitive scales and complaints.

The Chi square test or Fisher’s exact test (where expected values were less than 5) were used to compare categorical variables between groups. Student’s t test for independent samples was employed to compare continuous variables between two groups, when these were normally distributed. The normal distribution of cognitive variables was tested using the Kolmogorov-Smirnov’s test. Only the GDS variable did not present a normal distribution. In this case, the difference between the two age groups was assessed by Mann-Whitney’s non-parametric test. Pearson’s correlation coefficient was used to analyze the relationship among numeric variables. The level of significance adopted for the statistical tests was 5% (p<0.05). Version 6 of the SPSS statistical package was used for all statistical analyses.

## Results

A total of 67 individuals were interviewed, seven of whom were excluded for having completed higher level education. Other three individuals were excluded because they presented with depressive symptoms. This gave a final sample of 57 participants, 50 of whom were women.

Table 1 presents the socio-demographic data of the total sample. The sample was stratified into two age groups: Group 1, younger elderly, containing individuals aged between 56 and 74 years (n=35) and group 2, containing older elderly aged between 75 and 92 years (n=22). Comparisons between groups for categorical and continuous variables were performed. No significant difference was found between the groups for categorical variables (schooling and income). As expected, only a statistical difference for age was detected. The two age groups proved similar in terms of the socio-demographic profile.

**Table 1:** Socio-demographic profile of overall sample.

Variable	n=57	Standard deviation	%
Gender			
Female	50		87.7
Male	7		12.3
Age	57	71.39 (8.64)	100.0
Schooling			
Primary school unconcluded	34		59.64
Primary school concluded	23		0.4
Working			
Yes	11		19.3
No	46		80.7
Retired			
Yes	37		64.91
No	20		35.09
Personal income			
Up to 1 minimum wage	17		29.8
From 1 to 2 min. wages	16		28.1
From 2 to 3 min. wages	12		21.1
From 3 to 4 min. wages	6		10.5
From 4 to 5 min. wages	6		10.5

No statistical difference was found between Group 1 and Group 2 for performance on episodic and working memory tests or for overall cognitive performance. Similarly, no differences were seen between groups on the MAC-Q or the forgetfulness frequency scale as shown in Table 2.

**Table 2:** Results of comparisons between groups.

Variable	56 to 74 years Mean and SD	75 to 92 years Mean and SD	p-value
GDS	2.26 (1.31)	1.86 (1.49)	0.300^a^
MMSE	26.06 (2.87)	26.82 (2.59)	0.317^b^
FFS	11.94 (6.02)	11.32 (5.30)	0.692^b^
MAC-Q	23.46 (5.44)	22.91 (4.30)	0.691^b^
FIGURES	10.37 (2.64)	9.59 (3.08)	0.312^b^
DIGITS	14.44 (3.88)	14.91 (3.48)	0.648^b^

GDS: Geriatric Depression Scale; MMSE: Mini-Mental State Exam; FFS: Forgetfulness Frequency Scale; MAC-Q: Memory Complaint Questionnaire; FIGURES: Test with 18 figures; DIGITS: Sum of scores on forward and backward digit span.

a Mann-Whitney's U Test;

b Student's t test.

As depicted in Table 3, age was found not to be associated with the complaint or cognitive performance variables for the overall sample. However, significant correlations between cognitive variables and the two subjective memory measures (MAC-Q and the Forgetfulness Frequency Scale) were found, which was to be expected since these measures involve the same domain.

**Table 3:** Correlations between scores for cognitive variables and age.

		GDS	MMSE	FFS	MAC-Q	Figures	Digits
Age	Pearson p value n	-0.07 0.62 57	0.08 0.53 57	0.07 0.60 57	0.32 0.81 57	0.10 0.96 57	0.12 0.38 56
GDS	Pearson p value n		-0.24 0.08 57	0.50 0.75 57	0.77 0.58 57	-0.04 0.75 57	-0.19 0.16 56
MMSE	Pearson p value n			-0.99 0.47 57	-0.08 0.96 57	0.35 0.10 57	0.30 0.25 56
FFS	Pearsonp value n				0.43 0.01 57	-0.12 0.37 57	-0.07 0.97 56
MAC-Q	Pearson p value n					-0.122 0.38 57	-0.10 0.97 56
Figures	Pearson p value n						0.27 0.05 56

GDS; Geriatric Depression Scale; MMSE: Mini-Mental State Exam; FFS: Forgetfulness Frequency Scale; MAC-Q: Memory Complaint Questionnaire; FIGURES: Test with 18 figures; Dígits: Sum of scores on forward and backward digit span.

## Discussion

The aim of the present study was to recruit community-dwelling healthy elders who were participants at SESC, to ascertain any relationship between memory complaints and age, and between memory complaints and performance on cognitive tests in two different age groups. The results showed age not to be associated with any of the study variables. Cognitive performance in the two age group categories was statistically similar. Also, no significant difference was found between age groups in terms of memory complaints.

Although the literature reports decline in some memory modalities during the process of normal aging, including episodic and working memory, the present study found no significant correlation between age and cognitive variables, nor significant differences between the two age groups. The data suggest that the older adults recruited presented stability in the memory subsystems assessed. In other words, no age-related deficits, a typical finding in cross-sectional studies,^26^ was detected in our sample.

The findings of the present study are consistent with other such studies involving community-dwelling elders, in which memory complaints were shown to be unassociated with age.^9,27-29^

According to Germano-Neto,^2^ the notion that older elderly present greater memory complaints than younger elderly can be called into question, particularly when performance is preserved. The results reported contrast with those of the studies by Blazer and colleagues^30^ as well as Jonker and colleagues,^7^ who found a positive association between age and memory complaints.

Comparison of scores on complaint instruments (Forgetfulness frequency scales and MAC-Q) with the other variables revealed no significant correlations, with the exception of the correlation between the two scales assessing memory complaints, which revealed that subjects who reported a high frequency of forgetfulness also reported greater perceived memory impairment. Mattos and colleagues,^16^ in their study on a clinical population, also found a strong association between complaints reported to the interviewer and score on the MAC-Q.

The absence of an association in the present study between complaints and age or performance on cognitive tests may be explained by lifestyle, in that all participants had frequented SESC for many years. The older elderly were shown not to present worse cognitive performance, or to report greater memory complaints, thus constituting perhaps a particular sample. Participants in this study engaged in a range of physical, social, occupational and intellectual activities at the center such as yoga, swimming, handicrafts, drama workshops, intergenerational projects and discussion groups. This engagement may have contributed to the preservation of cognitive function in this group, described by several studies in healthy community elderly.^31-33^

Physical activity is considered a key factor in promoting health in general and has been identified as a strategy to prevent cognitive decline, and delay the onset of dementias.^32^ Other studies have also demonstrated the relationship between physical exercise and cognitive performance in the elderly.^34-36^ Wang and colleagues,^28^ in a community-based study, reported that intellectual, physical, social and leisure activities were positive factors toward maintaining good mental function among participants.

The data presented in the present study are consistent with the hypothesis that frequenting an organization such as SESC, which provides an opportunity to take part in a host of activities and engage socially, constitutes a protective factor against cognitive decline.

### Final considerations

It is important to note that this study is not subject to the characteristic bias of clinical and outpatient populations. Nevertheless, the sample studied is selective in as far as it only includes active elderly members of SESC. This group comprises elderly who are not ailing or presenting with severe handicaps, thus forming a group of “super” old which may not be representative of the Brazilian elderly population as a whole. Nevertheless, the data from this sample appears to suggest that engagement in physical, intellectual and social activities are conducive to good cognitive performance.

Future studies should further investigate the theme of memory complaints by verifying correlation with other variables such as schooling, gender, marital status, income and lifestyle, and elucidating the relationship between reported complaints and cognitive performance in the elderly population.
